# Plasma Epstein-Barr virus microRNA BART8-3p as a potential biomarker for detection and prognostic prediction in early nasopharyngeal carcinoma

**DOI:** 10.1038/s41598-024-58233-1

**Published:** 2024-03-28

**Authors:** Cheng Lin, Yuebing Chen, Xiandong Lin, Hewei Peng, Juan Huang, Shaojun Lin, Jianji Pan, Meifang Li, Jingfeng Zong

**Affiliations:** 1 Department of Radiation Oncology, Clinical Oncology School of Fujian Medical University, Fujian Cancer Hospital, Fuzhou, Fujian Province China; 2 Department of Radiation Biology, Fujian Medical University Cancer Hospital, Fujian Cancer Hospital, Fuzhou, Fujian Province China; 3 Department of Epidemiology and Health Statistics, Fujian Provincial Key Laboratory of Environment Factors and Cancer, School of Public Health, Fujian Medical University, Fuzhou, Fujian Province China; 4 Department of Radiation Oncology, Fujian Medical University Xiamen Humanity Hospital, Xiamen, Fujian Province China; 5 Department of Medical Oncology, Clinical Oncology School of Fujian Medical University, Fujian Cancer Hospital, Fuzhou, Fujian Province China

**Keywords:** Cancer screening, Head and neck cancer, Tumour biomarkers, Tumour virus infections

## Abstract

Epstein-Barr virus (EBV) encoded microRNA BART8-3p (miR-BART8-3p) was significantly associated with the metastasis in nasopharyngeal carcinoma (NPC). To explore the clinical values of plasma miR-BART8-3p in patients with early NPC. We retrospectively analyzed 126 patients with stage I and II NPC. A receiver operating characteristic curve was used to examine the diagnostic performance. Kaplan‒Meier analysis was applied to determine survival differences. Cox regression was used for univariate and multivariate analyses. Compared to healthy subjects, plasma EBV miR-BART8-3p was highly expressed in early NPC patients. The sensitivity, specificity, and area under the curve value of plasma miR-BART8-3p combined with plasma EBV DNA was up to 88.9%, 94.4%, and 0.931. Compared to patients with low expression of miR-BART8-3p, patients with high expression of miR-BART8-3p had poorer 5-year overall survival (OS) (98.9% vs. 91.1%, P = 0.025), locoregional recurrence-free survival (LRRFS) (100% vs. 83.9%, P < 0.001) and distant metastasis-free survival (DMFS) (98.9% vs. 88.0%, P = 0.006). Risk stratification analysis revealed that high-risk patients (with high levels of EBV DNA and miR-BART8-3p) had inferior OS, LRRFS, and DMFS than low-risk patients (without high levels of EBV DNA and miR-BART8-3p). Multivariate analysis verified that the high-risk group was an unfavorable factor for OS, LRRFS, and DMFS. A combination of plasma EBV miR-BART8-3p and EBV DNA could be a potential biomarker for the diagnosis and prognosis in early NPC.

## Introduction

Nasopharyngeal carcinoma (NPC) is prevalent in China and Southeast Asia, with a large unbalanced geographic global distribution^1^. Advances in radiotherapy technology, chemotherapy regimens, and staging systems have greatly improved the prognosis of NPC patients^2,3^. The 5-year survival rate of early NPC (stage I and II) has exceeded 90%. However, over 70% of patients are initially diagnosed with advanced stages (stage III and IV), as most early NPC cases are asymptomatic, leading to poor quality of life and survival^3^. Therefore, it is crucial to explore potential biomarkers to assist in the early diagnosis and treatment of NPC.

Epstein–Barr virus (EBV) infection is strongly related to NPC^4^. In epidemic areas, more than 95% of all NPC types are EBV-positive^5^. Numerous studies have reported that EBV-associated biomarkers, such as EBV-based antibodies and EBV DNA, have diagnostic value for NPC^6^. Nowadays, EBV DNA is considered a relatively mature method for NPC diagnosis in clinical practice. A meta-analysis revealed that the sensitivity and specificity of EBV DNA in the diagnosis of NPC were 75–78% and 92–96%, respectively^7^. However, EBV DNA has limited value in the detection of early NPC. Ji et al. reported that only 50% (7/14) of NPC patients who were positive for anti-EBV antibodies tested positive for EBV DNA at baseline^8^. In addition, EBV DNA can be affected by many factors, making its promotion and application difficult^9^. Therefore, novel biomarkers are urgently needed to improve diagnostic accuracy for early NPC.

EBV expresses two families of microRNA (miRNA), including BamHI A rightward transcripts (BARTs) and BHRF1. BARTs miRNA are supposed to be closely associated with the pathogenesis of NPC, including growth, metastasis, apoptosis, immune escape, and so on^10,11^. Accumulating evidence has demonstrated that BARTs miRNA are significantly overexpressed in NPC patients, suggesting that BARTs miRNA are potential biomarkers^12^. Our previous study revealed that EBV-encoded miR-BART8-3p was the most abundant BARTs microRNA in NPC tissues and was involved in the metastasis of NPC^13^. Moreover, plasma miR-BART8-3p was a novel biomarker in NPC patients^14^. However, whether circulating miR-BART8-3p could be useful in the detection and prognostic prediction of early NPC in endemic areas remains obscure. Herein, we aimed to evaluate the clinical significance of miR-BART8-3p in patients with early NPC and consequently assist precise medicine.

## Materials and methods

### Participants

One hundred and twenty-six early NPC patients were enrolled between September 2016 and May 2019. The inclusion criteria were as follows: (1) patients were histologically diagnosed with NPC, (2) NPC patients with stage I (T1N0M0) or II (T1N1M0 and T2N0-1M0), and (3) patients without any antitumor treatment before. The exclusion criteria were as follows: (1) patients who were lost to follow-up, and (2) patients with other types of cancer. A total of 142 healthy donor samples from our physical examination center were enrolled as controls, and they were matched with the NPC patients in terms of age and sex. The median age of healthy donors was 48.3 years, with 92 male patients and 50 female patients. Blood samples were collected after diagnosis but before any antitumor treatment. NPC patients were classified according to the American Joint Committee on Cancer (AJCC)/Union for International Cancer Control (UICC) 8th edition staging system. Overall survival (OS) was calculated from the day of diagnosis until death from any cause. Locoregional relapse-free survival (LRRFS) was calculated from the day of diagnosis to the time of locoregional recurrence. Distant metastasis-free survival (DMFS) was calculated from the day of diagnosis to the time of distant metastasis.

### Sample collection, RNA isolation, and cDNA conversion

The protocol for plasma sample collection and storage was previously described. In brief, blood samples were collected in ethylene diamine tetraacetic acid (EDTA) tubes. Tubes were stored at room temperature (15–25 °C) or 4 °C and processed within 1 h. Plasma was separated by centrifugation at 3000 × g for 10 min at 4 °C and then stored frozen at − 80 °C for further analysis. Total miRNAs were extracted from 200 μl plasma using the RNA miRNeasy Plasma Advanced Kit (Qiagen, Germany) according to the protocol recommended by the manufacturer. All plasma RNA samples were eluted in 20 μl of RNase-free water (Qiagen, Germany) and stored at − 80 °C for further processing. Reverse transcription of miRNA was performed using the TaqMan MicroRNA Reverse Transcription Kit (Applied Biosystems, Thermo Fisher Scientific, USA) with the following conditions: 16 °C for 30 min, 42 °C for 30 min, 85 °C for 5 min, and then kept at 4 °C.

### Quantitative analysis of miRNA

Quantitative polymerase chain reaction (q-PCR) was carried out using TaqMan Universal Master Mix II, no UNG (Applied Biosystems, Thermo Fisher Scientific, USA) and carried out on 7200 real-time PCR system (Applied Biosystems, Thermo Fisher Scientific, USA). The program for q-PCR involved 95 °C for 10 min, 45 cycles of 15 s at 95 °C, and 1 min at 60 °C. To estimate the absolute copy number of miRNA in plasma samples, a standard curve was established by quantitative PCR using serially diluted synthetic miRNA mimics. To adjust the efficiency of RNA extraction between samples, cel-miR-39 was added to plasma before miRNA extraction, and data from PCR amplification were normalized to Ce-miR-39 amplification according to the protocol of TaqMan Universal Master Mix II Kit. The specific information of TaqMan probes, primers for reverse transcription and qPCR were described in our previous study^14^. Multiple negative water blanks were included in each analysis. Two independent assays were performed. All determinations were repeated in triplicate. The information on miR-BART8-3p probes, primers, and synthetic miR-BART8-3p mimics was attached in Supplementary Table S1.

### EBV DNA isolation and quantitation

A total of 450 μl plasma was used for DNA extraction by a magnetic bead kit (catalog no. EA20160201; PerkinElmer), and then DNA was eluted by 60 μl nuclear-free water. EBV DNA concentrations were measured using a real-time quantitative PCR system (catalog no. DA-D065; Da An Gene) that amplified a DNA segment in the BamHI-W fragment region of the EBV genome. Data were collected and analyzed by ABI Prism 7500 Sequence Detector and 7500 Software (version 2.0.6; Applied Biosystems). The information on EBV DNA probes and primers was attached in Supplementary Table S1. The experimental procedures and operational details have been described in detail in an anterior study^15^.

### Statistical analysis

Data were processed and analyzed with SPSS 26.0, GraphPad Prism 9.4.1, and *R* software 4.2.1. The clinical characteristics were compared using chi-square tests for categorical variables. The Kaplan‒Meier (K–M) method was used to examine survival differences between the two groups, and differences were calculated by the log-rank test. Multivariable analyses were conducted with the Cox proportional hazards model. The Spearman correlation test was used to evaluate the correlations between miR-BART-8-3p levels and EBV DNA.

The cut-off value of plasma EBV DNA and miR-BART8-3p between high and low expression was defined by receiver operating characteristic (ROC) curves with Youden’s index using MedCalc software, version 20.123 (https://www.medcalc.org/). All assays were performed in duplicate. All the tests were 2-sided. *P* < 0.05 were considered statistically significant.

### Ethics approval and consent to participate

Our study was approved by the Ethical Review Committee of Fujian Cancer Hospital (approval no. 2015-010-02) and carried out according to relevant guidelines and regulations. All participants provided written informed consent for their blood to be sampled and analyzed.

## Results

### Circulating miR-BART8-3p was highly expressed in early NPC

**Figure 1:**
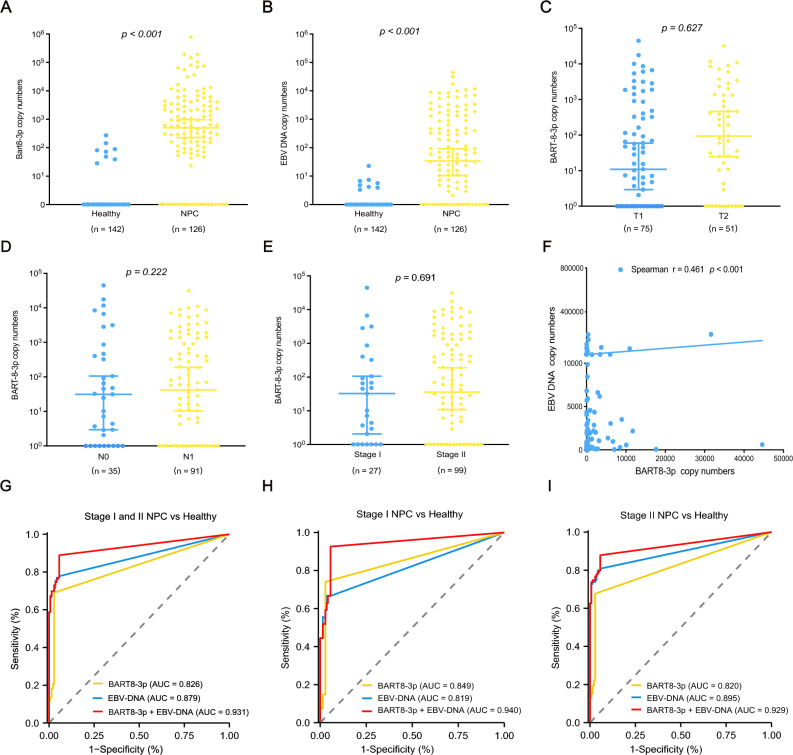
Diagnostic performance of plasma BART8-3p and EBV DNA in early nasopharyngeal carcinoma (NPC). Levels of plasma BART8-3p and EBV DNA in healthy controls and early NPC (**A**, **B**). Levels of BART8-3p in different T stages, N stages, and TNM stages (**C**–E). Correlation between expression of BART8-3p and EBV DNA (**F**). Receiver operating characteristic curves of miR-BART8-3p, EBV DNA, and the combination of miR-BART8-3p and EBV DNA (**G**–**I**).

With a median follow-up of 96.8 months, the 5-year overall survival (OS) rate, locoregional relapse-free survival (LRRFS) rate, and distant metastasis-free survival (DMFS) rate of early NPC were 96.8%, 94.7%, and 96.0%, respectively. The clinical characteristics of 126 early NPC patients are shown in Table 1. The median age was 49.0 years (range 15–84 years). The ratio of men to women was 1.86. The proportion of patients in stage II was higher than that in patients with stage I (78.6% vs. 21.4%). Compared to healthy volunteers, plasma miR-BART8-3p and EBV DNA were significantly increased in patients with early NPC (Fig. 1A, B). Further study revealed that the expression of miR-BART-8-3p was not significantly associated with the T stage, N stage, or clinical stage (Fig. 1C–E). Interestingly, there was a significant positive correlation between miR-BART8-3p and EBV DNA (Spearman r = 0.461; *P* < 0.001) (Fig. 1F).

**Table 1:** Variables of 126 patients with NPC grouped by plasma BART8-3p expression.

Variables	n (%)	Low expression (n = 92)	High expression (n = 34)	*P*
Age				0.528
< 60 years	101 (80.2)	75 (81.5%)	26 (76.5%)	
≧ 60 years	25 (19.8)	17 (18.5%)	8 (23.5%)	
Gender				0.957
Male	82 (65.1%)	60 (65.2%)	22 (64.7%)	
Female	44 (34.9%)	32 (34.8%)	12 (35.3%)	
Histology				1.000
NKUC	118 (93.7%)	86 (93.5%)	32 (94.1%)	
NKDC	8 (6.3%)	6 (6.5%)	2 (5.9%)	
ECOG score				0.294
0	122 (96.8%)	90 (97.8%)	32 (94.1%)	
1	4 (3.2%)	2 (2.2%)	2 (5.9%)	
T stage				0.185
T1	75 (59.5%)	58 (63%)	17 (50%)	
T2	51 (40.5%)	34 (37%)	17 (50%)	
N stage				0.517
N0	35 (27.8%)	27 (29.3%)	8 (23.5%)	
N1	91 (72.2%)	65 (70.7%)	26 (76.5%)	
TNM stage				0.264
I	27 (21.4%)	22 (23.9%)	5 (14.7%)	
II	99 (78.6%)	70 (76.1%)	29 (85.3%)	
LDH				0.611
Normal	130 (95.6)	89 (96.7%)	31 (91.2%)	
Elevated	6 (4.4)	3 (3.3%)	3 (8.8%)	

*NKUC* non-keratinizing undifferentiated carcinoma, *NKDC* non-keratinizing differentiated carcinoma, *ECOG* Eastern Cooperative Oncology Group, *LDH* lactate dehydrogenase.

Using 482.09 copies/ml and 1850.00 copies/ml as cut-off values for plasma miR-BART8-3p and EBV DNA, respectively, our data revealed that high expression of miR-BART8-3p was in 26.98% (34/126) of early NPC (Table 1). No significant differences were found between miR-BART8-3p and clinical features, including age, sex histology, ECOG score, TNM stage, and LDH.

### Diagnostic performance of plasma miR-BART8-3p

Further research showed that the sensitivity and specificity of miR-BART8-3p alone in the diagnosis of early NPC were 69.0% and 97.2%, respectively, with an AUC value of 0.826 (95% CI: 0.782–0.870) (Table 2, Fig. 1G). The sensitivity and specificity of EBV DNA alone in the diagnosis of NPC were 77.0% and 95.8%, respectively, with an AUC value of 0.879 (95% CI: 0.840–0.918). Interestingly, the AUC value reached 0.931 (95% CI: 0.901–0.961) when the combination of miR-BART8-3p and EBV DNA was considered (Fig. 1G). Of note, miR-BART8-3p had a superior sensitivity (74.1% vs. 66.7%) and AUC value (0.849 vs. 0.819) than EBV DNA in stage I NPC (Fig. 1H). While EBV DNA had a higher AUC value in stage II NPC (Fig. 1I).

**Table 2:** Diagnostic performance of plasma BART8-3p and EBV DNA in early NPC.

Comparison	SE (%)	SP (%)	PPV (%)	NPV (%)	AUC (95% CI)
EBV DNA					
Stage I vs Healthy	66.7	96.5	78.3	93.8	0.819 (0.726–0.914)
Stage II vs Healthy	79.8	95.8	92.9	87.2	0.895 (0.854–0.936)
Stage I and II vs Healthy	77.0	95.8	94.2	82.4	0.879 (0.840–0.918)
BART8-3p					
Stage I vs Healthy	74.1	97.2	83.3	95.2	0.849 (0.764–0.934)
Stage II vs Healthy	67.7	97.2	94.3	81.2	0.820 (0.770–0.869)
Stage I and II vs Healthy	69.0	97.2	95.6	78.0	0.826 (0.782–0.870)
EBV DNA plus BART8-3p					
Stage I vs Healthy	92.6	94.4	75.8	98.5	0.940 (0.886–0.994)
Stage II vs Healthy	87.9	94.4	91.6	91.8	0.929 (0.894–0.963)
Stage I and II vs Healthy	88.9	94.4	93.3	90.5	0.931 (0.901–0.961)

*SE* sensitivity, *SP* specificity, *PPV* positive predictive value, *NPV* negative predictive value, *AUC* Area Under Curve, *95% CI* 95% confidence interval.

### Complementation of miR-BART8-3p combined with EBV DNA

**Figure 2:**
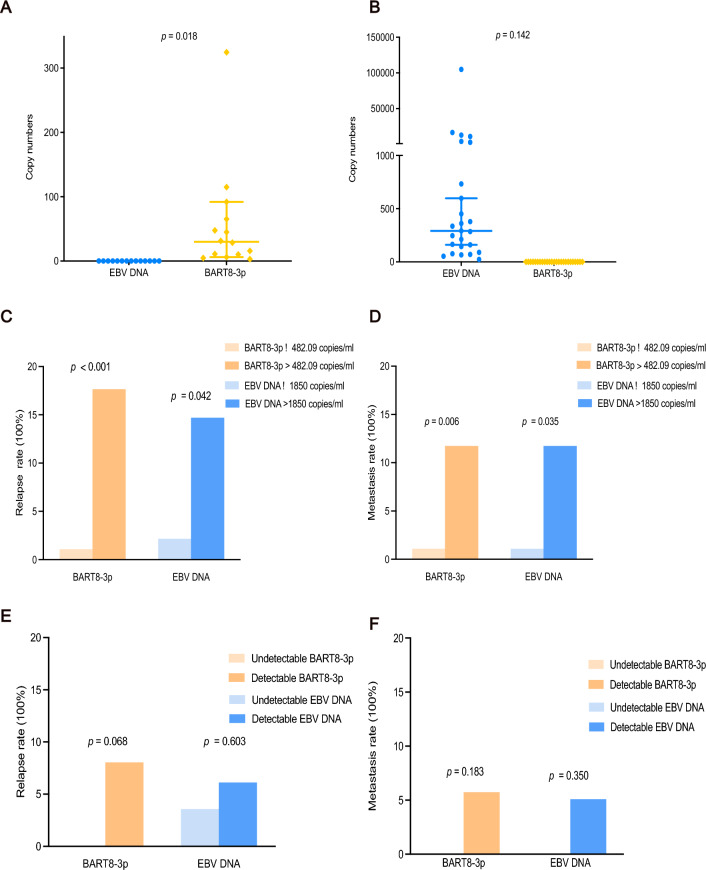
The complementary performance of BART8-3p and EBV DNA in early nasopharyngeal carcinoma (NPC). Patients with undetectable EBV DNA levels but detectable BART8-3p (**A**). Patients with undetectable BART8-3p but detectable EBV DNA (**B**). The performance of BATR8-3p and EBV DNA in predicting metastasis and relapse using different cut-off values (**C**–**F**).

To address the different and complementary roles between miR-BART8-3p and EBV DNA, further studies were examined. Our data revealed that 14 patients with EBV-DNA negative were miR-BART8-3p positive (Fig. 2A), whereas 25 patients with miR-BART8-3p negative were EBV DNA positive (Fig. 2B). Moreover, we found that patients with high levels of miR-BART8-3p were more likely to develop locoregional relapse than those with high levels of EBV DNA (Fig. 2C). And miR-BART8-3p had a higher power to distinguish patients who suffered from distant metastasis than EBV DNA (Fig. 2D). However, no significant difference of relapse rate between high and low expression of miR-BART8-3p was found using 0 copies/ml as the cut-off value of miR-BART8-3p (Fig. 2E, F).

Taken together, these findings suggest that miR-BART8-3p may serve as a promising biomarker for detecting early NPC and could be a complementary molecular marker in patients with EBV DNA negative.

### Prognostic value of plasma miR-BART8-3p in early NPC

**Figure 3:**
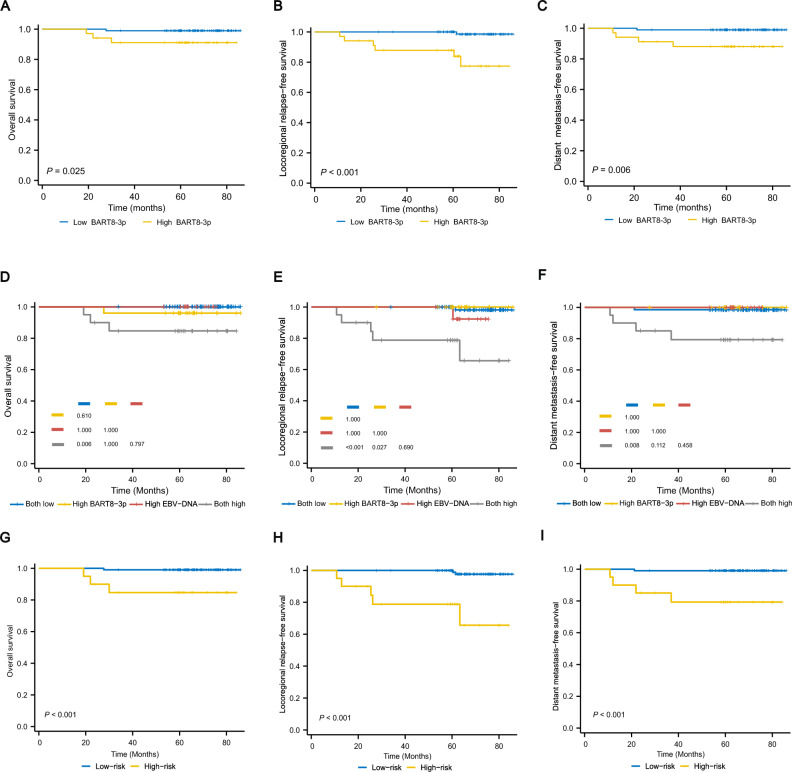
Kaplan‒Meier curves for overall survival (OS), locoregional relapse-free survival (LRRFS), and distant metastasis-free survival (DMFS) based on miR-BART8-3p in early nasopharyngeal carcinoma (NPC). Kaplan–Meier curves for OS, LRRFS, and DMFS according to low or high expression of BART8-3p (**A**–**C**). Survival outcomes of four subgroups based on EBV DNA and BART8-3p (**D**–**F**). Survival outcomes of patients at low- or high risk based on the combination of EBV DNA and BART8-3p (**G**–**I**).

Kaplan‒Meier analysis showed that compared to patients with low expression of miR-BART8-3p, patients with high expression of miR-BART-8-3p were correlated with significantly worse 5-year OS (98.9% vs. 91.1%, *P* = 0.025), LRRFS (100% vs. 83.9%, *P* < 0.001) and DMFS (98.9% vs. 88.0%, *P* = 0.006) (Fig. 3A–C, Table 3). In addition, we found that age had a significant influence on OS. The 5-year OS rate was 87.7% in patients older than 60 years, and 99.0% in those younger than 60 years (*P* = 0.027) (Table 3). Using 0 copies/ml as the cut-off value of miR-BART8-3p, we found that patients with detectable miR-BART8-3p levels were associated with poorer LRRFS. However, no significant differences were found between miR-BART8-3p levels and OS or DMFS (Supplementary Fig. S1).

**Table 3:** Univariate and Multivariate analysis of potential prognostic factors.

Characteristics	OS				LRRFS				DMFS			
	Univariate HR (95% CI)	P	Multivariate HR (95% CI)	P	Univariate HR (95% CI)	P	Multivariate HR (95% CI)	P	Univariate HR (95% CI)	*P*	Multivariate HR (95% CI)	*P*
Gender		0.544		–		0.258		–		0.484		–
Male	Reference		–		Reference		–		Reference		–	
Female	1.83 (0.26–13.02)		–		0.02 (0–15.29)		–		0.46 (0.51–4.09)		–	
Age(years)		**0.027**		**0.049**		0.723		–		0.967		–
< 60	Reference		Reference		Reference		–		Reference		–	
≥ 60	12.85 (1.34–123.59)		9.85 (1.01–95.86)		0.68 (0.08–5.66)		–		1.05 (0.12–9.38)		–	
T stage		0.189		–		0.576		–		0.371		–
T1	Reference		–		Reference		–		Reference		–	
T2	4.56 (0.47–43.80)		–		0.63 (0.12–3.23)		–		2.26 (0.38–13.54)		–	
N stage		0.893		–		0.428		–		0.697		–
N0	Reference		–		Reference		–		Reference		–	
N1	1.17 (0.12–11.23)		–		2.35 (0.28–19.55)		–		1.55 (0.17–13.83)		–	
TNM stage		0.868		–		0.658		–		0.940		–
I	Reference		–		Reference		–		Reference		–	
II	0.83 (0.09–7.93)		–		1.61 (0.19–13.40)		–		1.09 (0.12–9.74)		–	
LDH		0.082		–		0.212		–		0.110		–
Normal	Reference		–		Reference		–		Reference		–	
Elevated	7.47 (0.78–71.98)		–		3.93 (0.46–33.82)		–		5.98 (0.67–53.53)		–	
BART8-3p plus EBV DNA		**0.019**		**0.024**		** < 0.001**		** < 0.001**		**0.005**		**0.005**
Low-risk	Reference		Reference		Reference		Reference		Reference		Reference	
High-risk	20.71 (1.63–263.39)		13.71 (1.41–133.58)		19.57 (3.74–102.29)		19.57 (3.74–102.29)		23.59 (2.63–211.34)		23.59 (2.63–211.34)	

Significant values are in [bold].

*OS* overall survival, *LRRFS* locoregional recurrence-free survival, *DMFS* distant metastasis-free survival, *HR* hazard rate, *95% CI* 95% confidence interval, *LDH* lactate dehydrogenase.

### Risk stratification based on miR-BART8-3p combined with EBV DNA

To investigate whether the combination of miR-BART8-3p and EBV DNA can improve the prognostic capability in early NPC, patients were divided into four groups based on the levels of EBV DNA and miR-BART8-3p. Patients with both low levels of EBV DNA and miR-BART8-3p, high EBV DNA expression but low miR-BART8-3p expression, and high miR-BART8-3p expression but low EBV DNA expression had no significant differences in OS, LRRFS and DMFS (*P* > 0.05). However, subjects with high expression of both EBV DNA and miR-BART8-3p had the worst OS, LRRFS, and DMFS (*P* < 0.05) (Fig. 3D–F).

Given these findings, patients were classified into a high-risk group (patients with both high EBV DNA and high miR-BART8-3p) and a low-risk group (patients with neither high EBV DNA nor high miR-BART8-3p). The K-M analysis showed that the high-risk group had significantly worse OS, LRRFS, and DMFS than the low-risk group (Fig. 3G–I). Multivariate analysis verified that the high-risk group was an unfavorable factor for OS (HR 13.71; 95% CI, 1.41–133.58; *P* = 0.024), LRRFS (HR 19.57; 95% CI, 3.74–102.29; *P* < 0.001), and DMFS (HR 23.59; 95% CI, 2.63–211.34; *P* = 0.005) (Table 3).

## Discussion

Early detection of cancer is essential to increase survival rates, as approximately 50% of cancers are discovered at an advanced stage^16^. Nowadays, early identification of cancers using liquid biopsies remains a difficult challenge, as well as in NPC^17^. EBV-encoded BART miRNAs represent promising biomarkers in NPC^6,18–20^. Studies have reported that miR-BART8-3p was highly expressed in NPC patients, and it promoted metastasis and radioresistance by regulating NF-κB and Erk1/2 and ATM/ATR signaling pathway, highlighting its promising value as a biomarker^13,21^. In this article, we found that plasma miR-BART8-3p was a potential biomarker for diagnosis and prognosis in early NPC. The combination of miR-BART8-3p and EBV DNA significantly improved the power in detecting early NPC. Moreover, the risk stratification model based on miR-BART8-3p and EBV DNA was an independent prognostic factor for OS and DMFS in early NPC.

Early screening of NPC could significantly improve NPC patient outcomes using plasma EBV DNA^22^. Accumulating data have reported that the sensitivity of EBV DNA screening for early NPC patients was approximately 76–81.5%^8,20^, which is consistent with our study^14^. The sensitivity of miR-BART8-3p in early NPC was lower than that in nonmetastatic NPC patients (92.0%) in our previous study. The reason may be due to lower tumor loads and earlier tumor stages. Although the sensitivity of miR-BART8-3p was lower than that of EBV DNA, the specificity of miR-BART8-3p was higher than that of EBV DNA, especially in stage I NPC. More importantly, plasma miR-BART8-3p can be positive in EBV DNA-negative NPC patients. The combination of miR-BART8-3p and EBV DNA could provide a higher sensitivity of 88.9% and, a higher specificity of 88.9%, with an AUC value of 0.931. Therefore, miR-BART8-3p is a potential biomarker for detecting early NPC and could be a complementary or alternative tool in patients with negative EBV DNA.

Of note, studies have suggested that the clinical values of BARTs miRNA could be different in NPC patients. Gao et al. reported that miR-BART8 and BART10-3p had the best performance in identifying recurrent NPC in patients with undetectable plasma EBV DNA, and BART19-5p had the highest performance in identifying NPC with undetectable serological EBV DNA^23^. Octavia Ramayanti showed that vesicle-bound EBV-BART13-3p could distinguish NPC from head and neck cancer and asymptomatic EBV infections^19^. Other studies also reported that BART7, BART13-3p, and BART2-5p had valuable roles in the detection of NPC, with AUC values larger than 0.9^18,20^. The inconsistency between the above articles might be due to different extraction and detection methods and samples used. In addition, different cut-off values of BART miRNAs may also account for the discordance. Thus, choosing suitable biomarkers may be the top priority in clinical practice in NPC^24^. Further study is needed to improve the diagnostic ability of miR-BART8-3p and to develop an optimal detection strategy for early NPC.

EBV miR-BART8-3p was reported to promote metastasis of NPC, indicating that miR-BART8-3p is a worthy biomarker for metastasis^13,21^. Of note, T stage, N stage, or TNM stage was not significantly associated with patients’ outcomes in our study, which may account for the limited sample size and small differences in survival time in early NPC patients. Our study found that high levels of miR-BART8-3p were related to a higher risk of distant metastasis in early NPC, which was consistent with our previous study in nonmetastatic NPC. Importantly, miR-BART8-3p had a better capacity to predict recurrence and metastasis than EBV DNA. In addition, the risk model and multivariate analysis revealed that high-risk patients (both high expression of miR-BART8-3p and EBV DNA) were significantly associated with inferior OS, LRRFS, and DMFS. Therefore, for patients with high levels of miR-BART8-3p and EBV DNA, more aggressive treatment regimens might be recommended. Besides, more caution should be paid to the monitoring of recurrence and metastasis in early NPC. In the future, personalized and precise strategies based on the levels of miR-BART8-3p are urgently needed.

Our study had a few limitations. First, the generalizability of the results to nonendemic areas is uncertain since we only studied individuals in NPC endemic areas. Second, the extraction and detection procedures and methods, including centrifugation speed and endogenous control, should be modified to improve the sensitivity and specificity. Third, our study was a retrospective single-center study with a small sample and had no independent validation cohort, which could not avoid potential selection bias. The values of plasma miR-BART8-3p in early NPC need to be confirmed by prospective, multicenter, and large-sample studies.

## Conclusion

Plasma miR-BART8-3p was a promising biomarker for diagnosing and predicting patients with early NPC, and the prognostication could be enhanced by combination with EBV DNA.

### Supplementary Information

Supplementary Information.

## Data Availability

The data generated in this study are available within the article and its Supplementary Data files. Detailed data generated in this study are available upon request from the corresponding author.
