# Supplementary material for: Plasma Epstein-Barr virus microRNA BART8-3p as a potential biomarker for detection and prognostic prediction in early nasopharyngeal carcinoma
Source: Sci Rep. 2024 Mar 28;14:7433. doi: 10.1038/s41598-024-58233-1 (PMC10978918; doi:10.1038/s41598-024-58233-1)
Supplement: Supplementary file 1 — Supplementary Information. [file 41598_2024_58233_MOESM1_ESM.docx]

**Supplemental Material**


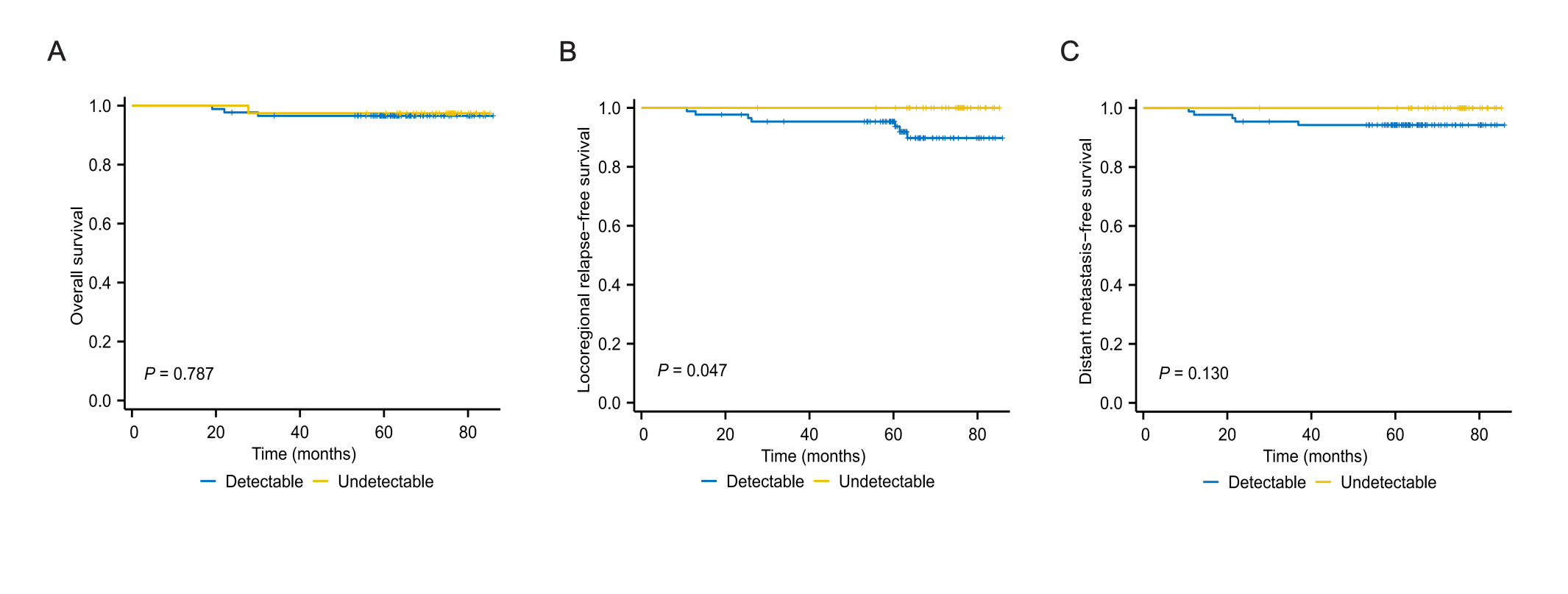


**Supplemental Figure S1** Kaplan‒Meier curves for overall survival (A), locoregional relapse-free survival (B), and distant metastasis-free survival (C) using 0 copies/ml as the cut-off value of miR-BART8-3p.

| **Category** | **Sequence** |
| --- | --- |
| BART8-3p TaqMan Probe | 5’- CGCACTGGATACGACTCTACG - 3’ |
| BART8-3p Forward primer | 5’- ATCGTCACAATCTATGGGGT-3’ |
| BART8-3p Reverse primer | 5’- GCAGGGTCCGAGGTATTC-3’ |
| BART8-3p Reverse transcription primer | GTCGTATCCAGTGCAGGGTCCGAGGTATTCGCACTGGATACGACTCTACG |
| Synthetic BART8-3p mimics | 5’- GUCACAAUCUAUGGGGUCGUAGA-3’ |
| EBV DNA Forward Primer | 5’-TGCCAAAGAGCCAGATCTAAGG-3’ |
| EBV DNA Reverse primer | 5’-AAAGTGTCAGATTTTGGGTCCAA-3’ |
| EBV DNA Probe | 5’-FAM-CAGCCCCAAAGCGGGTGCAGTAAC-BHQ1-3’ |
| EBV DNA Reverse transcription primer | 5’-GTCGTATCCAGTGCAGGGTCCGAGGTATTCGCACTGGATACGACCCCTGG-3’ |

**Supplementary Table 1** The sequence of the forward primers, reverse primers, probe, and synthetic mimics.
